# Wildlife associates of nine‐banded armadillo (*Dasypus novemcinctus*) burrows in Arkansas

**DOI:** 10.1002/ece3.8858

**Published:** 2022-05-15

**Authors:** Brett A. DeGregorio, John T. Veon, Andrhea Massey

**Affiliations:** ^1^ U.S. Geological Survey Fish and Wildlife Cooperative Research Unit University of Arkansas Fayetteville Arkansas USA; ^2^ Department of Biological Sciences University of Arkansas Fayetteville Arkansas USA

**Keywords:** burrow commensals, ecosystem engineer, nine‐banded armadillo, rufugia, wildlife monitoring

## Abstract

The Nine‐banded Armadillo (*Dasypus novemcinctus*) is a widespread burrowing species with an expanding geographic range across the southeastern and midwestern United States. Armadillos dig numerous, large burrows within their home ranges and these burrows are likely used by a diverse suite of wildlife species as has been reported for other burrowing ecosystem engineers such as Gopher Tortoises (*Gopherus polyphemus*), Desert Tortoises (*Gopherus agassizi*), and Black‐tailed Prairie Dogs (*Cynomys ludovicianus*). We used motion‐triggered game cameras at 35 armadillo burrows in 4 ecoregions of Arkansas and documented 19 species of mammals, 4 species of reptile, 1 species of amphibian, and 40 species of bird interacting with burrows. Bobcat (*Lynx rufus*), Coyote (*Canis latrans*), Eastern Cottontail (*Sylvilagus floridanus*), Gray Fox (*Urocyon cinereoargenteus*), Gray Squirrel (*Sciurus carolinensis*), Northern Raccoon (*Procyon lotor*), Virginia Opossum (*Didelphis virginiana*), and unidentified rodents (mice and rats) were documented using burrows in all four ecoregions. We documented wildlife hunting, seeking shelter, rearing young in, and taking over and modifying armadillo burrows. The rate of use was highest in the Mississippi Alluvial Valley, a landscape dominated by agriculture, where natural refugia may be limited and rodents are abundant. Armadillo burrows are clearly visited and used by numerous wildlife species to fulfill various life stage requirements, and this list will likely expand if more attention is devoted to understanding the role of armadillos burrows. Armadillos are important ecosystem engineers, and their ecological role warrants more investigation and attention as opposed to only being viewed and managed as agricultural and garden pests.

## Graphical abstract

Nine‐banded Armadillos dig extensive burrow systems. These burrows provide refuge for numerous other wildlife species. This article explores the array of wildlife that use armadillo burrows and describes how different animals interact with burrows.

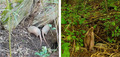

## 1 INTRODUCTION

There are numerous descriptions in the ecology literature of the astounding diversity of invertebrates and vertebrates associated with Gopher Tortoise (*Gopherus polyphemus*) burrows. Over 300 species have been documented using these extensive burrows (Dziadzio & Smith, 2016; Jackson & Milstrey, 1989; Kent et al., 1997). Because of the extraordinary number of species that seek shelter in tortoise burrows, the Gopher Tortoise is rightfully lauded as an ecosystem engineer and keystone species that must be protected for the health of the ecosystem. However, the Gopher Tortoise is not the only burrow excavator in the southeastern United States. The Nine‐banded Armadillo (*Dasypus novemcinctus*: hereafter; armadillo) has an extensive and expanding range in the southeastern and midwestern United States and can be regularly found in the forests, grasslands, and bottomlands of 15 states (Feng & Papeş, 2015). Because individual armadillos excavate and maintain up to 10 burrows within their territory, these refugia occur in remarkably high densities (up to 27 per ha; Platt et al., 2004). Additionally, these burrows are large in size allowing them to be potentially used by numerous other vertebrates. Our goal here was to use motion‐triggered game cameras to document the vertebrates associated with armadillo burrows in four distinct ecoregions of Arkansas, USA.

Structurally, the burrows of nine‐banded armadillos are approximately 20 cm wide by 15 cm in height and are on average 50 cm deep but have been measured as long as 4.5 m (Clark, 1951; McDonough et al., 2000). Armadillos excavate their own burrows and disperse the soil at the front of the burrow creating “aprons.” Although not as extensive as the sandy aprons of Gopher Tortoise burrows, the entrance of armadillo burrows can include mounded soil, leaf litter, or exposed ground. Both the burrows themselves as well as the exposed soils and leaf litter at the entrances of armadillo burrows are used by a wide array of wildlife species (Butler, 2020; Clark, 1951; Lamb et al., 2020). For some species, these burrows serve as refugia from predators or the elements and can be used for sleeping, resting, or birthing. For others, the depth and length of the burrows provide thermal refugia during hot summer months or cold winter periods. Other species are likely attracted to the burrows because they forage for the insects, reptiles, or small mammals residing within the burrows.

The wildlife species that utilize armadillo burrows likely vary based on geographic location. Understanding how burrow use varies geographically provides information about the value of armadillo burrows to the wildlife community in general. In areas where subterranean refugia are limited or absent, armadillo burrows may represent valuable and sought‐after refuges. In other areas where alternative retreat sites are available (areas with rocky outcrops and crevices), armadillo burrows may not be as important and may be less frequently used. Our objectives here were to (1) document the vertebrate species that use armadillo burrows over the course of a calendar year in four distinct ecoregions of Arkansas, USA, where mammal communities and land cover all vary substantially, (2) compare patterns in usage rates and species usage patterns between the ecoregions, and (3) document and describe the different ways in which wildlife use armadillo burrows.

## 2 MATERIALS AND METHODS

### 2.1 Study sites

We conducted this study in four ecoregions in Arkansas, USA, including the Ozark Mountains, the Mississippi Alluvial Valley, the Ouachita Mountains, and the Gulf Coastal Plain. In the Ozarks, we studied burrow use at two sites. The first was Hyland Park, a 28‐ha woodlot within a suburban neighborhood in the city of Fayetteville, Arkansas. Hyland Park is heavily forested with a mixed oak‐hickory composition similar to much of the forest cover across the Ozark Mountains. The substrate is clay and rock and the site is bisected by numerous spring‐fed creeks and several caves and rock outcrops are present which can serve as refugia for wildlife. Our second study site in the Ozark Mountain Ecoregion was Bear Hollow Nature Preserve located approximately 60 km East of Fayetteville. Bear Hollow is a 160‐ha natural area that is covered by mixed oak‐hickory forest and is steeply sloped with numerous rocky outcrops. The site is bisected by Rockhouse Creek and is surrounded on all sides by additional forested set‐aside areas.

In the Mississippi Alluvial Valley, we studied the use of armadillo burrows at Cache River National Wildlife Refuge (NWR). The Mississippi Alluvial Valley is a flat and predominantly sandy area devoid of exposed rock or rock outcroppings. While extensive bottomland hardwood forests once existed in the area, much of this land has been converted to commercial agriculture including rice and soybeans. The area of Cache River NWR where we deployed cameras consisted of old field habitat and levees surrounding oxbow wetlands and was approximately 250 ha in size.

In the Ouachita Mountains, we monitored burrow use at one study site, a 33‐ha private property that runs along the Cossatot River and resides on the city lines of De Queen and Gillham, Arkansas. The property is heavily forested, dominated by pine‐hardwood, and mixed oak forests. This site contains a mixture of river bottoms and mountainous terrain (elevation range: 125–165 m). This property is bisected by a spring‐fed creek and is further divided into a northern and southern compound by a county road. It should also be noted that many of the neighboring properties are in timber management, consisting of short rotation of shortleaf pine (*Pinus echinata*).

In the Gulf Coastal Plain, we monitored armadillo burrow use on three private properties that each consisted of low vegetation due to grazing and surrounded by mixed pine‐hardwood forests. The first was a 202‐ha rural cattle pasture in Fulton, Arkansas. The second site was a 198‐ha rural cattle pasture outside of the city limits of Texarkana, Arkansas. The third site was a 17‐ha pasture just outside the eastern edge of the city limits of Texarkana, Arkansas USA, and just 3.6 km southwest of our third site. These sites lacked exposed rock outcrops or caves that serve as natural refugia.

### 2.2 Burrow monitoring

Beginning in March 2020, we located burrows by walking the sites and opportunistically encountering burrows. We selected burrows where we felt cameras would not attract attention from hikers or recreators and where the field of view of the burrow was relatively unobstructed by vegetation. At selected burrows, we set motion‐triggered wildlife cameras. We used several types of cameras for this study (Reconyx Microfire, Bushnell HD Aggressor, Bushnell Core, and Browning Strike Force HD). All cameras were set to take a burst of 3 photos each time they were triggered and to have a reset period between 0 and 8 s (depending on the available options of the camera model) before triggering again. Cameras were placed on nearby trees or tripods placed between 2 and 3 m from the burrow entrance. Cameras were set approximately 50–75 cm off the ground and angled toward the burrow entrance to ensure we captured all wildlife interacting with the burrows. While we attempted to standardize the distance from the camera to each burrow, this varied due to topography, orientation of each burrow, and the presence/absence of vegetation. We acknowledge that cameras set further back from burrows or with vegetation in the foreground may record fewer detections of small animals such as birds, rodents, and reptiles. The amount of time that individual cameras were left in place varied from 1 month to slightly over a year due to various factors including camera failures, flooding, and making accommodations to landowners. All cameras were removed from the field by June 2021.

We downloaded memory cards from cameras approximately once per month. We used Timelapse 2.0 (Greenberg et al., 2019) to review all photographs and assign species ID to each wildlife trigger. We combined all photographs taken within a 5‐min period as a single detection to reduce the likelihood of double‐counting individuals (DeGregorio et al., 2021). For each wildlife detection, we identified the species present, the number of individuals visible, and we recorded which part of the burrow an animal interacted with including passing by (no interaction with burrow), apron (animal interacting with the mounded sand, bare ground, or leaf litter piled in front of the burrow), entrance (animals sniffing, foraging, or inspecting the opening to the burrow), or interior (animals that moved beyond the entrance of a burrow to enter, exit, or investigate the tunnel of the burrow). We also extracted the date and time of all interactions with burrows. While we attempted to identify each trigger to the species level, for most rodents we categorized them simply as “mice” or “rats.” For all other vertebrates, we excluded detections where we were unable to identify the species.

For the 10 mammal species most frequently observed interacting with armadillo burrows, we calculated the interaction rate or frequency of interaction for each ecoregion. We focused on these mammals because they were most frequently detected interacting with burrows, whereas most bird species that were frequently detected were more typically observed foraging in the burrow area rather than entering burrows. We defined interaction rate as the total number of detections of that species at a burrow divided by the number of camera days collected for that burrow. We excluded all instances of animals that passed by the burrow without interacting with it and only included instances in which the animal visibly interacted with the apron, entrance, or interior of the burrow itself.

## 3 RESULTS

Excluding 3 cameras lost to flooding or malfunction, we deployed cameras at 35 armadillo burrows for a total of 5879 camera days. We monitored burrows from a minimum of 26 days to a maximum of 399 days. The number of burrows monitored as well as camera days collected within each ecoregion varied (Table 1).

**TABLE 1:** List of study sites and number of Nine‐banded Armadillo (*Dasypus novemcinctus*) burrows monitored with motion‐triggered game cameras in Arkansas, USA

Study site	Ecoregion	No. of burrows monitored	No. of camera days	No. of wildlife detections
Cache River National Wildlife Refuge	Mississippi Alluvial Valley	6	1950	8740
Hyland Park	Ozark Mountains	7	1529	4172
Bear Hollow Natural Area	Ozark Mountains	8	863	562
DeQueen	Ouachita Mountains	7	1100	1301
Hope and Texarkana	Gulf Coastal Plains	6	437	1344

### 3.1 Wildlife species at armadillo burrows

**FIGURE 1:**
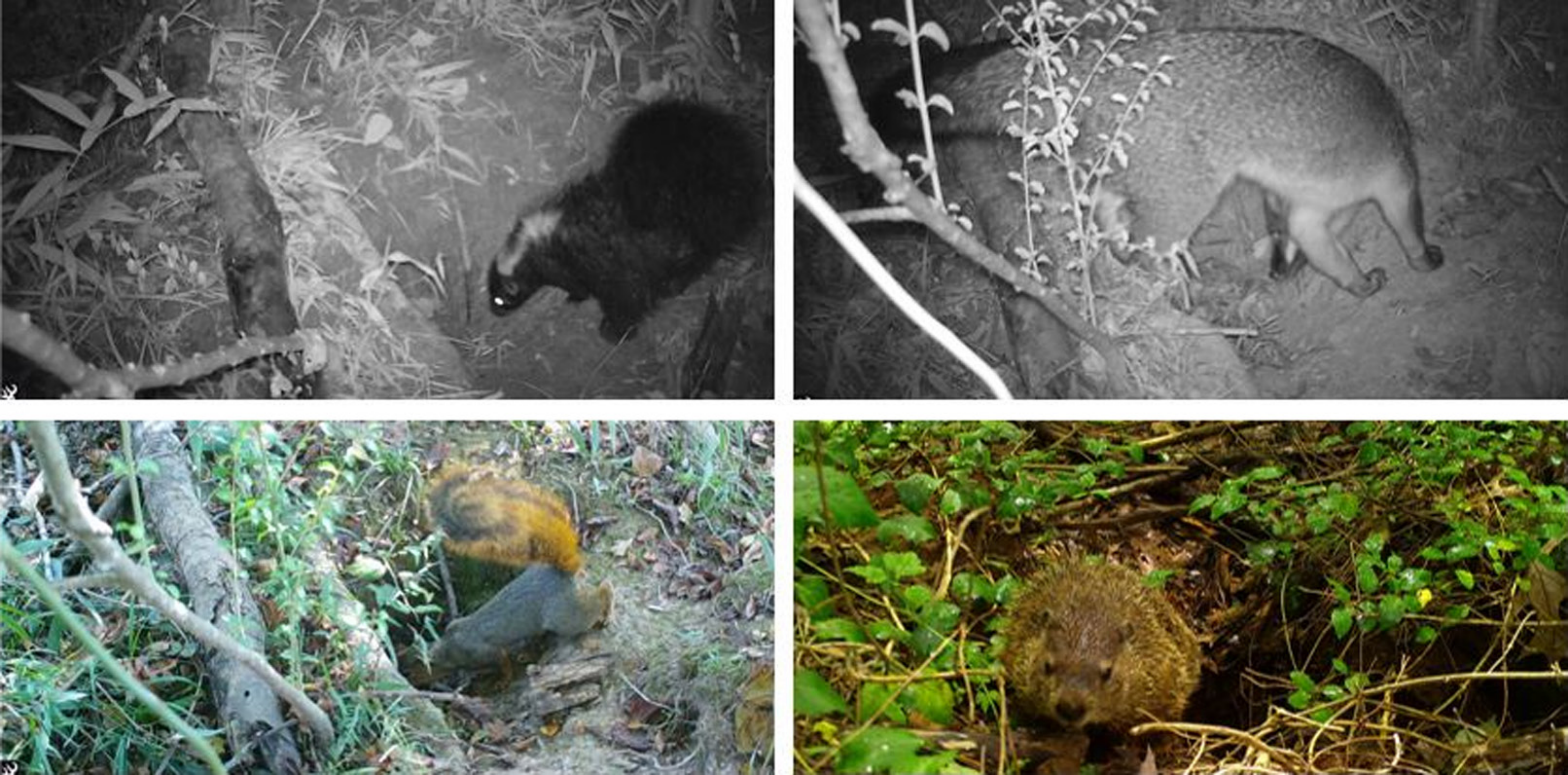
During a calendar year of monitoring Nine‐banded Armadillo (*Dasypus novemcinctus*) burrows in Arkansas, we documented 23 species of mammal interacting with burrows including Striped Skunks (*Mephitis mephitis*: top left), Gray Fox (*Urocyon cinereoargenteus*: top right), Fox Squirrels (*Sciurus niger*: bottom left), and Groundhogs (*Marmota monax*: bottom right). Photographs by Brett A. DeGregorio (bottom right) and John Veon (all others)

We recorded a total of 16,119 wildlife detections occurring at, in, or around armadillo burrows. Most detections (93%) were from mammals (Table 2). We identified 23 species of mammals interacting or passing by armadillo burrows (Figure 1). We also captured numerous mice and rats that we were unable to identify to species although we suspect that most rats belonged to the genus *Rattus* and most mice belonged to the genus *Peromyscus*. We also documented 7 species of reptile, 2 species of amphibian, and 41 species of bird in the vicinity of burrows. Overall, we documented 19 species of mammals, 4 species of reptile, 1 species of amphibian, and 40 species of bird directly interacting with or entering/exiting armadillo burrows.

**TABLE 2:** Wildlife documented passing by or interacting with nine‐banded armadillo (*Dasypus novemcinctus*) burrows in Arkansas, USA

Species	No. of detections	Ecoregions	Burrow interactions
Mammals			
Bobcat (*Lynx rufus*)	57	gcp, mav, ozm, oum	apron, entrance, interior, pass
Cow (*Bos taurus*)	9	gcp	apron, entrance, pass
Coyote (*Canis latrans*)	48	gcp, mav, ozm, oum	apron, entrance, pass
Domestic Cat (*Felis catus*)	47	ozm, oum	apron, pass
Domestic Dog (*Canis lupus familiaris*)	7	gcp, mav	entrance, pass
Eastern Chipmunk (*Tamias striatus*)	606	ozm	apron, entrance, interior
Eastern Cottontail (*Sylvilagus floridanus*)	325	gcp, mav, ozm, oum	apron, entrance, pass
Eastern Woodrat (*Neotoma floridana*)	288	gcp, mav, ozm	apron, entrance, interior, pass
Fox Squirrel (*Sciurus niger*)	382	gcp, mav	apron, entrance, interior, pass
Gray Fox (*Urocyon cinereoargenteus*)	32	gcp, mav, ozm, oum	apron, entrance, interior, pass
Gray Squirrel (*Sciurus carolinensis*)	3043	gcp, mav, ozm, oum	apron, entrance, interior, pass
Groundhog (*Marmota monax*)	9	ozm	apron, entrance, interior
Long‐tailed Weasel (*Mustela frenata*)	1	gcp	pass
Muskrat (*Ondatra zibethicus*)	1	mav	pass
Nine‐banded armadillo (*Dasypus novemcinctus*)	1953	gcp, mav, ozm, oum	apron, entrance, interior, pass
North American Beaver (*Castor canadensis*)	4	mav	pass
Raccoon (*Procyon lotor*)	2432	gcp, mav, ozm, oum	apron, entrance, interior, pass
Red Fox (*Vulpes vulpes*)	72	ozm, oum	apron, entrance, interior, pass
Southern Flying Squirrel (*Glaucomys volans*)	3	mav, oum	apron, pass
Striped Skunk (*Mephitis mephitis*)	114	gcp, mav	apron, entrance, interior, pass
Virginia Opossum (*Didelphis virginiana*)	1672	gcp, mav, ozm, oum	apron, entrance, interior, pass
White‐tailed Deer (*Odocoileus virginianus*)	1001	gcp, mav, ozm, oum	apron, entrance, pass
Wild Hog (*Sus scrofa*)	9	gcp, mav, oum	pass
Mouse sp.	2424	gcp, mav, ozm, oum	apron, entrance, interior, pass
Rat sp.	513	mav, ozm	apron, entrance, interior, pass
Amphibians			
Eastern Spotted Newt (*Notophthalmus viridescens*)	1	mav	apron
Green Frog (*Lithobates clamitans*)	1	ozm	pass
Frog sp. (*Lithobates* sp)	5	gcp, mav, ozm, oum	apron, pass
Reptiles			
Western Ratsnake (*Pantherophis obsoletus*)	1	mav	interior
North American Racer (*Coluber constrictor*)	1	mav	apron
Eastern Garter Snake (*Thamnophis sirtalis*)	1	gcp	pass
Speckled Kingsnake (*Lampropeltis holbooki*)	1	gcp	interior
Five‐lined Skink (*Plestiodon fasciatus)*	29	gcp, mav, oum	pass, apron, interior
Three‐toed Box Turtle (*Terrapene carolina triunguis*)	3	mav, oum, ozm	pass
Red‐eared Slider (*Trachemys scripta*)	1	mav	pass
Birds			
American Crow (*Corvus brachyrhynchos*)	1	ozm	apron
American Robin (*Turdus migratorius*)	98	mav, ozm	apron, pass
Blue Jay (*Cyanocitta cristata*)	36	gcp, mav, ozm	apron, pass
Brewer's Blackbird (*Euphagus cyanocephalus*)	1	ozm	apron
Brown‐headed Cowbird (*Molothrus ater*)	1	gcp	pass
Brown Thrasher (*Toxostoma rufum*)	32	gcp, mav, ozm	apron, entrance
Carolina Chickadee (*Poecile carolinensis*)	13	gcp, mav, ozm, oum	apron
Carolina Wren (*Thryothorus ludovicianus*)	355	gcp, mav, ozm, oum	apron, entrance, interior, pass
Common Yellowthroat (*Geothylpis trichas*)	3	mav	apron
Dark‐eyed Junco (*Junco hyemalis*)	48	mav, ozm	apron
Eastern Towhee (*Pipilo erythrophthalmus*)	16	mav	apron
Fox Sparrow (*Passerella iliaca*)	1	mav	apron
Gray Catbird (*Dumetella carolinensis*)	30	mav, ozm	apron
Hermit Thrush (*Catharus guttatus*)	7	ozm	apron
House Finch (*Haemorhous mexicanus*)	10	gcp, oum	apron, entrance, pass
House Sparrow (*Passer domesticus*)	2	gcp	apron
House Wren (*Troglodytes aedon*)	2	oum	apron, pass
Indigo Bunting (*Passerina cyanea*)	1	mav	apron
Kentucky Warbler (*Geothlypis Formosa*)	2	gcp	apron
Mallard (*Anas platyrhynchos*)	59	mav	apron
Mourning Dove (*Zenaida macroura*)	9	mav, ozm	apron
Northern Cardinal (*Cardinalis cardinalis*)	355	gcp, mav, ozm, oum	apron, pass
Northern Flicker (*Colaptes auratus*)	30	mav, ozm	apron
Northern Mockingbird (*Mimus polyglottos*)	1	gcp	apron
Ovenbird (*Seiurus aurocapilla*)	12	mav	apron
Pileated Woodpecker (*Dryocopus pileatus*)	10	mav, ozm	apron
Pine Warbler (*Setophaga pinus*)	1	gcp	apron
Prothonotary Warbler (*Protonotaria citrea*)	2	mav	apron
Red‐shouldered Hawk (*Butea lineatus*)	5	gcp, mav, ozm	apron, entrance
Red‐bellied Woodpecker (*Melanerpes carolinus*)	12	mav	apron
Red‐tailed Hawk (*Buteo jamaicensis*)	2	mav	apron, entrance
Ruby‐crowned Kinglet (*Corthylio calendula*)	1	mav	apron
Rusty Blackbird (*Euphagus carolinus*)	1	mav	apron
Swainson's Thrush (*Catharus ustulatus*)	20	ozm	apron
Tufted Titmouse (*Baeolophus bicolor*)	12	gcp, ozm, oum	apron, entrance, pass
Turkey Vulture (*Cathartes aura*)	17	mav	apron
White‐breasted Nuthatch (*Sitta carolinensis*)	4	ozm	apron
White‐throated Sparrow (*Zonotrichia albicollis*)	183	mav, ozm	apron, entrance
Wild Turkey (*Meleagris gallopavo*)	17	oum, ozm	apron, pass
Wood Duck (*Aix sponsa*)	50	mav	apron
Yellow‐breasted Chat (*Icteria virens*)	4	mav	apron

Abbreviations: GCP, gulf coastal plain; MAV, Mississippi alluvial valley; OUM, Ouachita mountain ecoregion; OZM, Ozark mountain ecoregion.

### 3.2 Regional differences in burrow use

The number of mammal species documented at armadillo burrows varied slightly between ecoregions with 17 mammal species documented at burrows in the Gulf Coastal Plain, 19 in the Mississippi Alluvial Valley, 16 in the Ozark Mountains, and 14 in the Ouachita Mountains. Excluding feral or free‐roaming domestic species, the richness for each ecoregion was 15 (Gulf Coastal Plain), 16 (Mississippi Alluvial Valley), 15 (Ozark Mountains), and 13 (Ouachita Mountains).

We documented several patterns where species were only documented in particular ecoregions. For instance, Eastern Chipmunks (*Tamias striatus*) were only documented in the Ozark Mountains where they frequently used armadillo burrows for foraging and dustbathing. Within Arkansas, the Eastern Chipmunk is absent from the Gulf Coastal Plain and Mississippi Alluvial Valley (Sasse, 2003) but is found throughout the Ouachita Mountains. However, there were patterns in burrow use for more widespread species such as the Groundhog (*Marmota monax*) which occurs throughout Arkansas but was only documented using burrows in the Ozark Mountains (*N* = 9). The Striped Skunk is also a widespread species occurring throughout Arkansas but was only detected using armadillo burrows in the Gulf Coastal Plain and Mississippi Alluvial Valley. Other notable absences include the Red Fox (*Vulpes vulpes*) in the Gulf Coastal Plain and Mississippi Alluvial Valley, Fox Squirrels (*Sciurus niger*) from both mountain ecoregions, and Eastern Woodrats (*Neotoma floridana*) from burrows in the Ouachita Mountains. The Long‐tailed Weasel (*Mustela frenata*), a species of greatest conservation need in Arkansas, was only documented at a burrow in the Gulf Coastal Plain and appeared to be passing by the burrow and not directly interacting with it. Most other differences in species occurrence were for species passing by burrows rather than those interacting with or using burrows and are not explored in depth here. Species that used armadillo burrows and were documented in all four ecoregions were Bobcat (*Lynx rufus*), Coyote (*Canis latrans*), Eastern Cottontail (*Sylvilagus floridanus*), Gray Fox (*Urocyon cinereoargenteus*), Gray Squirrel (*Sciurus carolinensis*), Raccoon (*Procyon lotor*), Virginia Opossum (*Didelphis virginiana*), mice and, unsurprisingly, Nine‐banded Armadillo (*Dasypus novemcinctus*).

**FIGURE 2:**
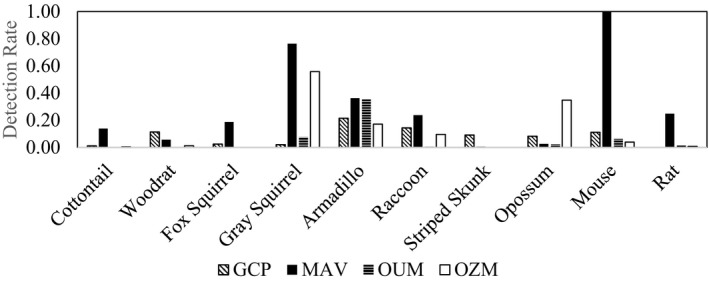
The interaction rate (number of burrow interactions divided by the number of camera days) of commonly observed mammals at Nine‐banded Armadillo (*Dasypus novemcinctus*) burrows in four ecoregions of Arkansas. GCP, Gulf Coastal Plains; MAV, Mississippi Alluvial Valley; OUM, Ouachita Mountains; OZM, Ozark Mountains

We calculated the detection rate for all mammals combined within each of the ecoregions by dividing the total number of detections by camera days. We only included detections by mammals that were interacting with burrows and excluded all instances in which animals were simply passing by. The Mississippi Alluvial Valley had the highest interaction rate with 3.21 animal interactions with burrows per day. The Ozark Mountains had 1.56 interactions per day followed by the Gulf Coastal Plain (0.87) and then the Ouachita Mountains (0.59). The interaction rate by individual species varied between ecoregions (Figure 2). Of particular note was the high interaction rate of small mammals including Gray Squirrels (*Sciurus carolinensis*), Eastern Cottontail (*Sylvilagus floridanus*), Fox Squirrel (*Sciurus niger*), mice, and rats with burrows in the Mississippi Alluvial Valley. Also, the Virginia Opossum used burrows in the Ozark Mountains at more than twice the frequency than in other ecoregions (Figure 2).

### 3.3 Wildlife interactions with armadillo burrows

Approximately 25% of the animals we detected on our cameras were passing by the armadillo burrows without visibly interacting or acknowledging them. Approximately 68% of animals interacted with the apron area of the burrow. In 3% of detections, the animal interacted with the entrance to the burrow. We documented animals foraging in this area, collecting spiderweb from the entrance, sniffing and investigating the burrow, or sometimes even urine marking the entrance of the burrow. On 3% of detections, animals entered the armadillo burrows. We documented Bobcats (*Lynx rufus*), Eastern Chipmunks (*Tamis striatus*), Eastern Woodrats (*Neotomo floridana*), Eastern Fox Squirrels (*Sciurus niger*), Gray Squirrels (*Sciurus carolinensis)*, Gray Fox (*Urocyon cinereoargenteus*), Red Fox (*Vulpes vulpes*), Groundhogs (*Marmota monax*), Raccoons (*Procyon lotor*), Virginia Opossum (*Didelphis virginiana*), Striped Skunks (*Mephitis mephitis*), mice, rats, and Nine‐banded Armadillos entering or exiting burrows. Additionally, we documented Western Ratsnakes (*Pantherophis obsoletus*), Speckled Kingsnakes (*Lampropeltis holbrooki*), and Five‐lined Skinks (*Plestiodon fasciatus*) entering and exiting burrows. Relatively few birds were observed entering burrows; however, Carolina Wrens (*Thryothorus ludovicianus*) were observed numerous times entering completely into burrows in addition to frequently foraging for insects inside and around the entrance to burrows.

### 3.4 Interesting anecdotes

**FIGURE 3:**
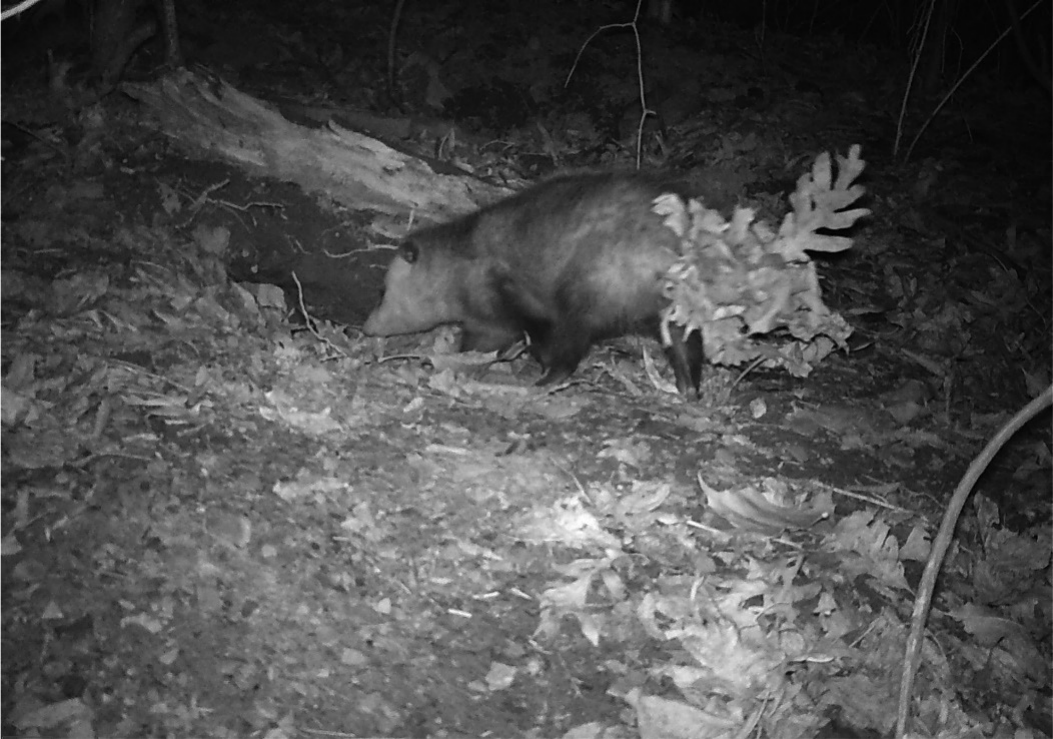
A Virginia Opossum (*Didelphis virginiana*) carries a bunch of dried leaves in its prehensile tail. As it enters the Nine‐banded Armadillo (*Dasypus novemcinctus*) burrow directly in front of it, the opossum will drop the leaves forming a plug at the entrance of the burrow Photograph by Brett A. DeGregorio

**FIGURE 4:**
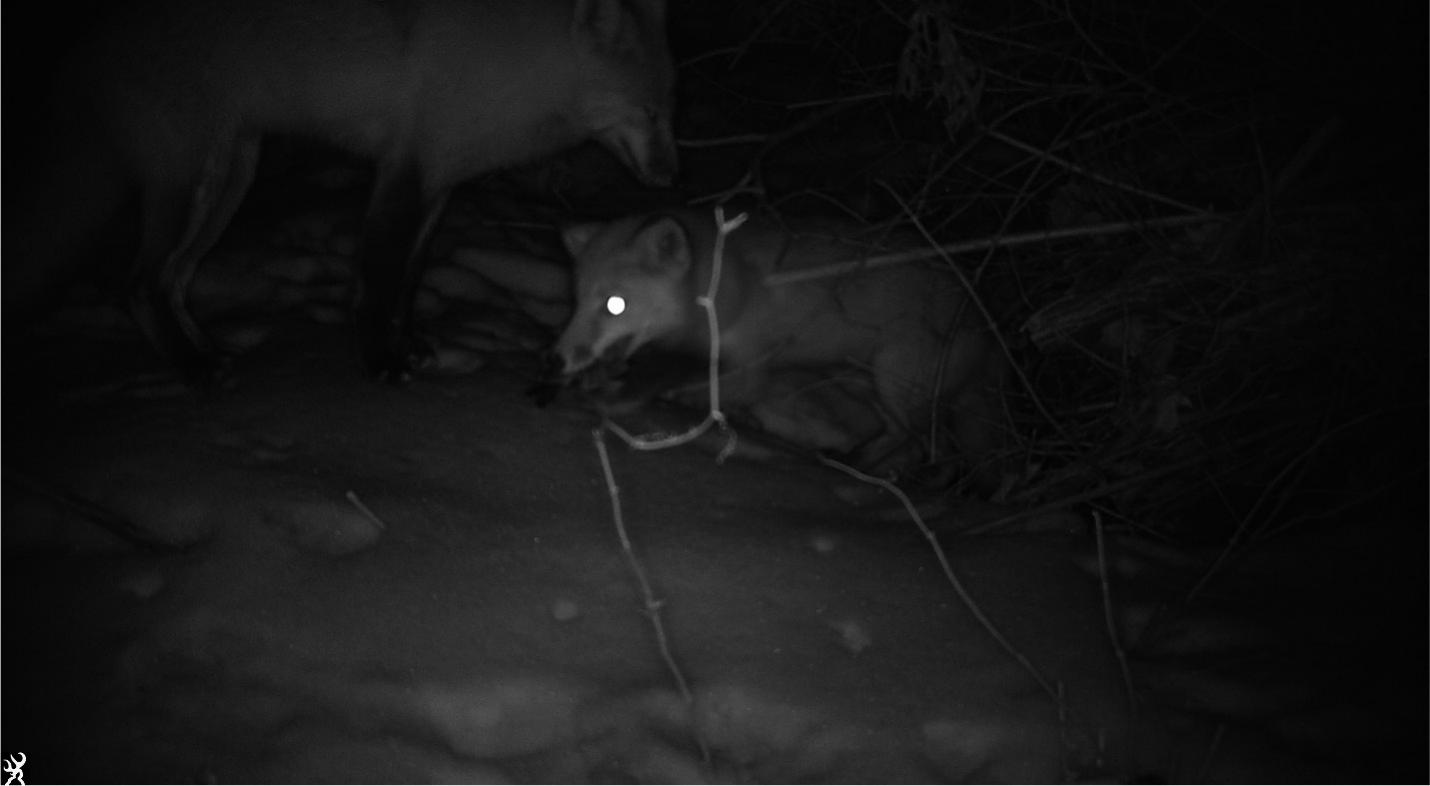
Some wildlife, such as these Red Fox (*Vulpes vulpes*) will take over Nine‐banded Armadillo (*Daspus novemcinctus*) burrows, modify their size and structure, and use them as dens. This Red Fox pup, eating an American Robin (*Turdus migratorius*), appears to have been born inside this modified armadillo burrow in the Ozark Mountains of Arkansas. Photograph by Brett A. DeGregorio

**FIGURE 5:**
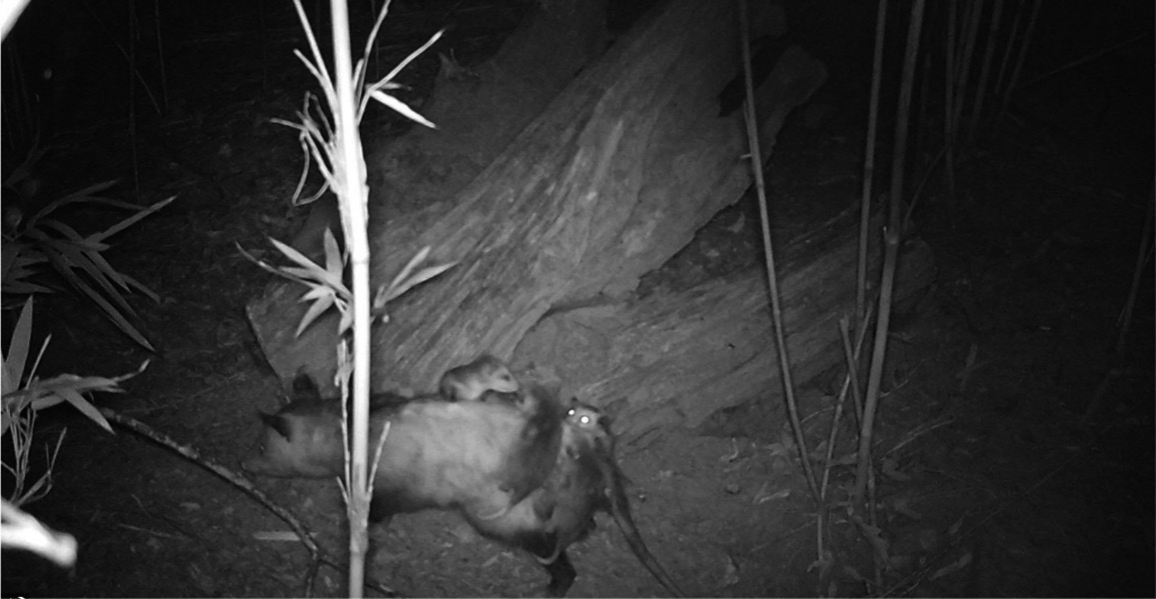
Virginia Opossum (*Didelphis virginiana*) appear to use Nine‐banded Armadillo (*Dasypus novemcinctus*) burrows as retreat sites when caring for their young during transition stages, a time when both mother and offspring are vulnerable. This Opossum entered an armadillo burrow with a visibly full pouch and emerged one week later with these babies clinging to her back. Photograph by Andrhea Massey

**FIGURE 6:**
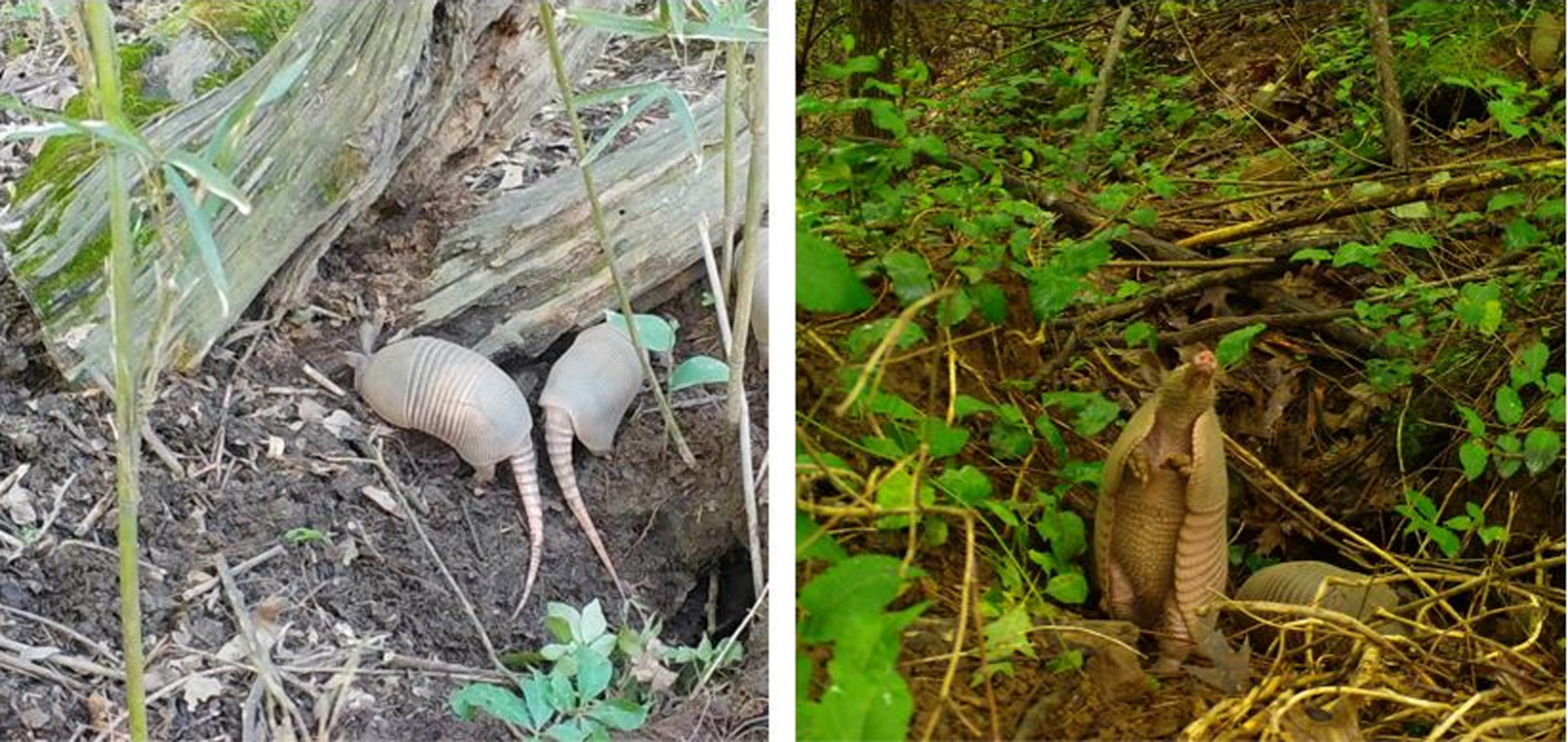
Nine‐banded Armadillos (*Dasypus novemcinctus*) are born inside burrows and monitoring of these burrows can provide information about the timing of reproduction. Photographs by Brett A. Degregorio

**FIGURE 7:**
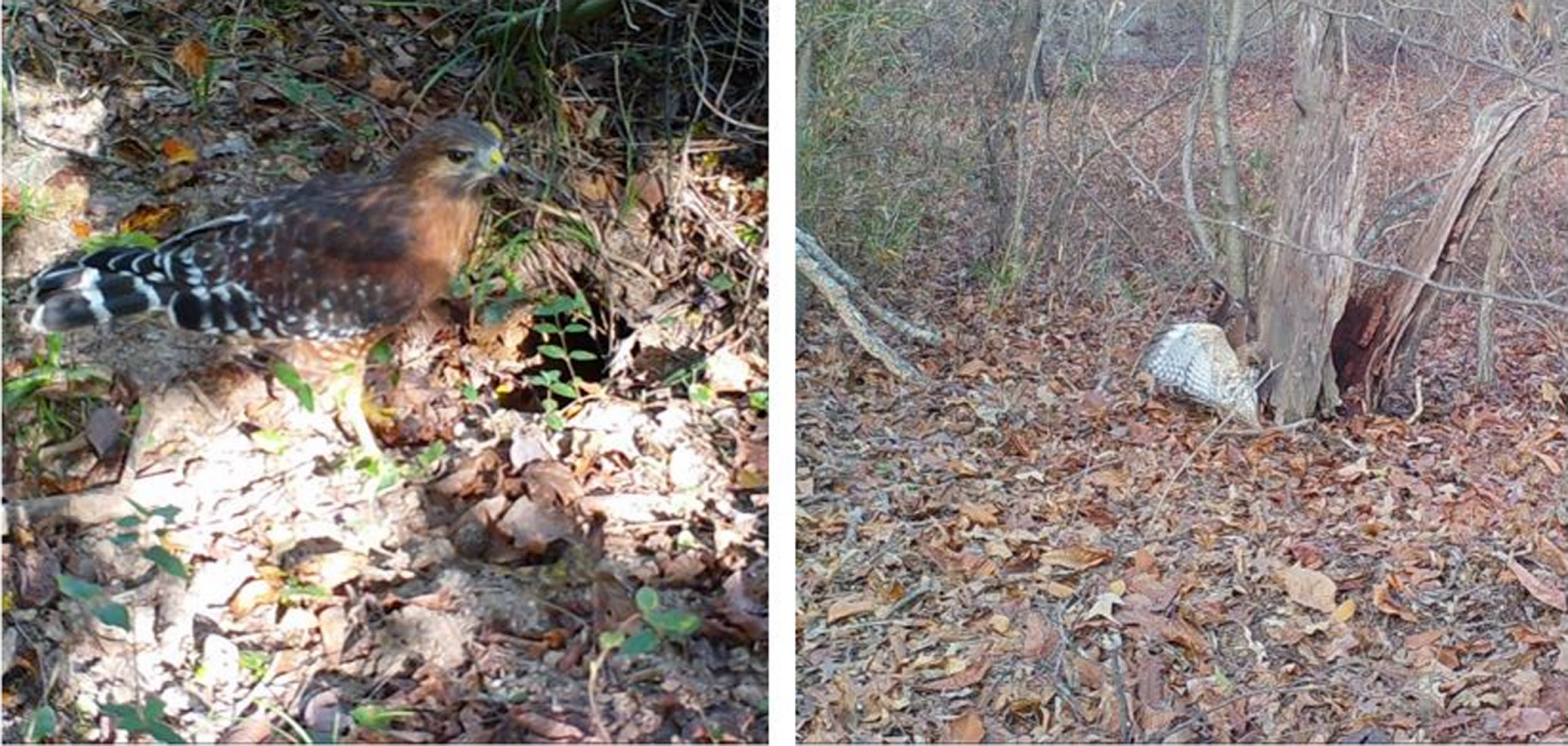
Red‐shouldered hawks (*Buteo lineatus*) frequently hunted frogs, snakes, and lizards at the entrances to Nine‐banded Armadillo (*Dasypus novemcinctus*) burrows. Photographs by Andrhea Massey

**FIGURE 8:**
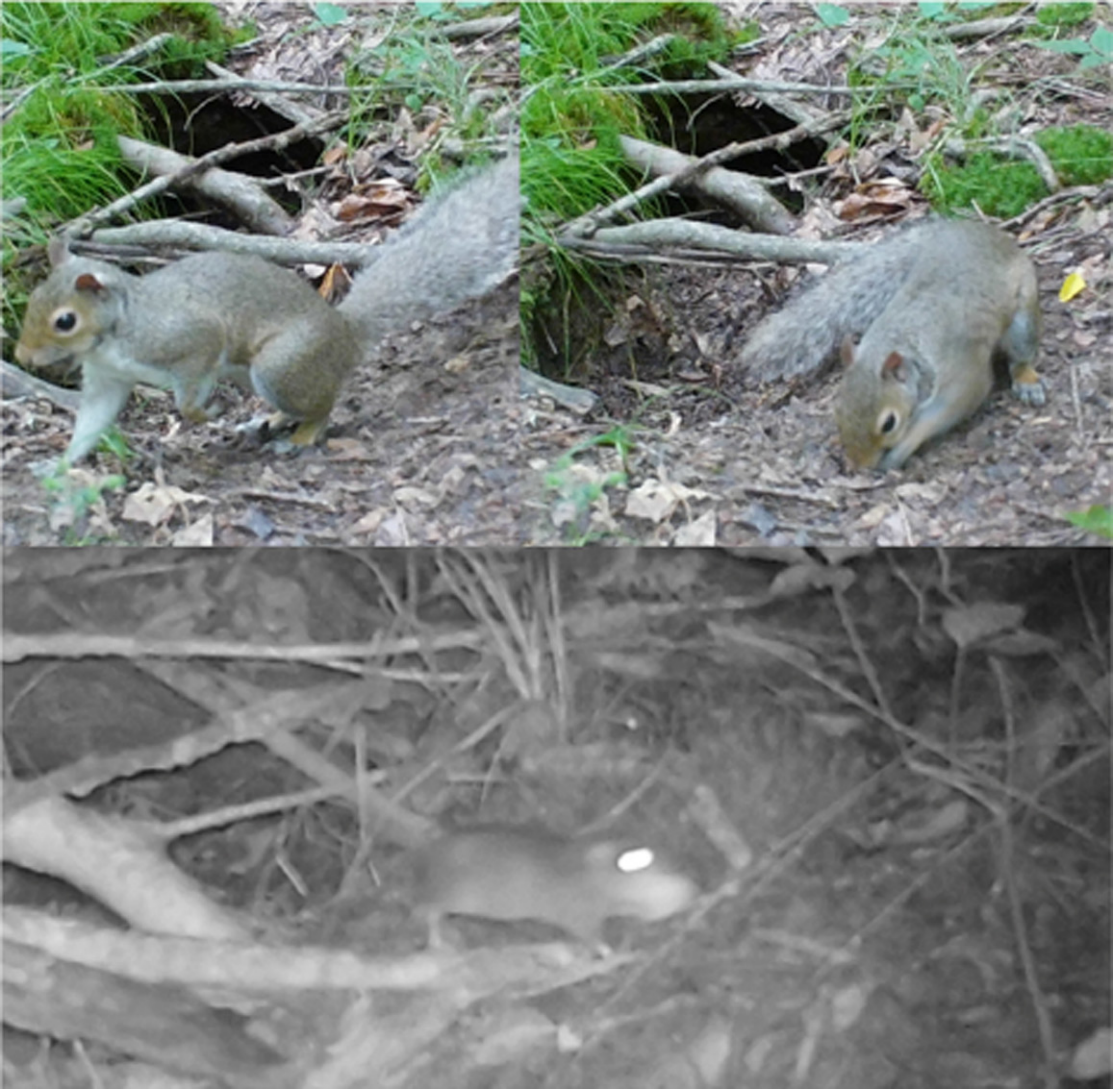
Gray Squirrel (*Sciurus carolinensis*) caching acorns and using those food stores at a later date. Also pictured is a mouse who discovered a squirrel cache and took acorn. Photographs by John Veon

1. Virginia Opossums frequently utilized armadillo burrows. We recorded 148 detections where opossum interacted with the entrance or interior of armadillo burrows and another 799 interactions with burrow aprons. On several occasions, Virginia Opossum were documented carrying large bunches of dried leaves with their prehensile tails. As they entered armadillo burrows, they would place the leaves at the entrance of the burrow obscuring and partially plugging the entrance (Figure 3). We observed adult opossum displaying this behavior as well as juveniles doing this with adults suggesting this is a learned behavior. This may be a strategy to increase insulation within burrows during the cold winter months when Virginia Opossum are vulnerable to frostbite (Blumenthal & Kirkland, 1976).
2. While most wildlife species used armadillo burrows without modifying them, we observed both Groundhogs and Red Fox taking over and modifying the burrows for their own long‐term use. This modification consisted of expanding the entrance and, for Red Fox, digging a second entrance chamber. Armadillos were observed later using the burrow modified by the Groundhog, but no armadillo was documented using the burrow after it was taken over by the Red Fox. Interestingly, the Red Fox used this burrow to give birth to a pup (Figure 4).
3. Virginia Opossum also appeared to use armadillo burrows as safe places when caring for offspring (Figure 5). We observed several Virginia Opossum spending long stretches of time (up to 7 days) in burrows when caring for vulnerable neonates. It was unclear if opossum gave birth in the armadillo burrows or entered them with a pouch full of very young opossum and remained in the burrows until they were larger and visible clinging to the mother's back upon exit.
4. Armadillos also gave birth in their burrows (Figure 6). Monitoring burrow entrances provided life history information such as the timing of reproduction. The young armadillos were often seen during the day foraging around the entrances of their burrows for 3–7 days before following their mother to a different burrow.
5. Armadillo burrows may be important foraging areas for raptors (Figure 7). We observed both Red‐tailed Hawks (*Buteo jamaicensis*) and Red‐shouldered Hawks (*Buteo lineatus*) capturing prey at the entrances of burrows. In bottomland habitats, frogs, lizards, and snakes were often observed using armadillo burrows, creating hunting opportunities for Red‐shouldered Hawks. Similarly, mice were frequent users of armadillo burrows, particularly in the Mississippi Alluvial Valley where our cameras detected at least one mouse per day per burrow creating an opportunity for Red‐tailed Hawks.
6. Armadillo burrows may also be important food banks or landmarks for food storage (Figure 8). We observed Gray Squirrels (*Sciurus carolinensis*) frequently caching food and digging up previously cached food near or on the apron of burrows. We also observed mice digging up and taking away the acorns buried by squirrels.

## 4 DISCUSSION

Here, we documented a wide range of wildlife species using the burrows of the Nine‐banded Armadillo for various purposes such as shelter, foraging, hiding food, reproduction, or foraging (Table 2). Numerous investigators have studied the wildlife associated with the burrows of Gopher Tortoises, Prairie Dogs, and Desert Tortoises (e.g., Agha et al., 2017; Dziadzio & Smith, 2016; Tyler & Shackford, 2002). Other species of armadillos such as the Giant Armadillo (*Priodontes maximus*) excavate burrows that have been shown to be used by numerous other vertebrate species (Desbiez & Kluyber, 2013). Each new investigation adds species to the list and enforces the value of these ecosystem engineer species to the wildlife community (Sun et al., 2021). In some areas of the United States, armadillos are considered introduced species and are viewed negatively due to real or perceived threats to human health and economics. Our hope is that this investigation adds to the growing body of literature showing the value of armadillo burrows to other wildlife species (e.g., Butler, 2020; Lamb et al., 2020) and helps to shift the sentiment of armadillos from nuisances to important components of the ecosystem. Currently, armadillos are expanding their geographic range northwards and where established, their burrows can be quite dense (Feng & Papeş, 2015; Platt et al., 2004). The burrows they create provide complexity to their environment and are used by many other wildlife species.

We predicted that armadillo burrows might be used by more species and more frequently in parts of Arkansas where natural retreat sites such as caves and rock crevices are absent or rare. In Arkansas, this would primarily be in the Mississippi Alluvial Valley and Gulf Coastal Plain where soils are sandy, rock is rare or absent, and topography is flat. In the Mississippi Alluvial Valley, the interaction rate of wildlife with burrows was very high indicating that this might be the case. However, the high interaction rate was strongly influenced by mice and rats (Figure 2). These animals may be at elevated densities due to widespread commercial agriculture in the area (White et al., 2012) and the high numbers of mice and rats creates hunting opportunities for native raptors and mammals.

We did observe variation in use by ecoregion with Virginia Opossum and Groundhogs more likely to use burrows in the Ozark Mountains (Table 2; Figure 2). This may be a consequence of opossums seeking thermal refugia more frequently in this montane climate. Opossums are prone to mortality from frostbite (Blumenthal & Kirkland, 1976) and their persistence in northern climates likely hinges on availability of retreat sites such as burrows of other animals or anthropogenic structures (Kanda & Fuller, 2005; Kanda et al., 2009). The geographic expansion of armadillos northward may benefit opossum that are already living in thermally challenging environments. Groundhogs are widespread in Arkansas but were only documented using armadillo burrows in the Ozarks. This may be due to the difficulty of excavating burrows in this rocky environment.

In addition to understanding the benefits of armadillos to co‐occurring wildlife, monitoring of armadillo burrows with game cameras may benefit researchers by providing an efficient way to sample the wildlife community. Because numerous species of wildlife interact with these burrows for various purposes, cameras stationed at these wildlife hotspots could increase efficiency in documenting mammal communities without the need to use lures or bait to increase detection rates. Armadillo burrows are likely important components of the environment for numerous reasons and both a diverse array of both wildlife and researchers can benefit from their presence.

## CONFLICT OF INTEREST

None of the authors have conflicts of interest to report.

## AUTHOR CONTRIBUTIONS

**Brett A. DeGregorio:** Conceptualization (lead); Data curation (lead); Formal analysis (lead); Funding acquisition (lead); Investigation (lead); Methodology (lead); Project administration (lead); Writing – original draft (lead). **John T. Veon:** Data curation (supporting); Formal analysis (supporting); Investigation (supporting); Methodology (supporting); Writing – original draft (supporting); Writing – review & editing (supporting). **Andrhea Massey:** Data curation (supporting); Formal analysis (supporting); Investigation (supporting); Methodology (supporting); Writing – original draft (supporting); Writing – review & editing (supporting).

## Data Availability

Data have been uploaded and are available via Dryad https://doi.org/10.25338/B82050.
